# Model-based analysis of pattern motion processing in mouse primary visual cortex

**DOI:** 10.3389/fncir.2015.00038

**Published:** 2015-08-05

**Authors:** Dylan R. Muir, Morgane M. Roth, Fritjof Helmchen, Björn M. Kampa

**Affiliations:** ^1^Laboratory of Neural Circuit Dynamics, Brain Research Institute, University of ZürichZürich, Switzerland; ^2^Biozentrum, University of BaselBasel, Switzerland; ^3^Department of Neurophysiology, Institute of Biology II, RWTH Aachen UniversityAachen, Germany

**Keywords:** mouse, primary visual cortex (V1), pattern integration, plaid stimuli, model-based analysis, Bayesian framework, two-photon imaging

## Abstract

Neurons in sensory areas of neocortex exhibit responses tuned to specific features of the environment. In visual cortex, information about features such as edges or textures with particular orientations must be integrated to recognize a visual scene or object. Connectivity studies in rodent cortex have revealed that neurons make specific connections within sub-networks sharing common input tuning. In principle, this sub-network architecture enables local cortical circuits to integrate sensory information. However, whether feature integration indeed occurs locally in rodent primary sensory areas has not been examined directly. We studied local integration of sensory features in primary visual cortex (V1) of the mouse by presenting drifting grating and plaid stimuli, while recording the activity of neuronal populations with two-photon calcium imaging. Using a Bayesian model-based analysis framework, we classified single-cell responses as being selective for either individual grating components or for moving plaid patterns. Rather than relying on trial-averaged responses, our model-based framework takes into account single-trial responses and can easily be extended to consider any number of arbitrary predictive models. Our analysis method was able to successfully classify significantly more responses than traditional partial correlation (PC) analysis, and provides a rigorous statistical framework to rank any number of models and reject poorly performing models. We also found a large proportion of cells that respond strongly to only one stimulus class. In addition, a quarter of selectively responding neurons had more complex responses that could not be explained by any simple integration model. Our results show that a broad range of pattern integration processes already take place at the level of V1. This diversity of integration is consistent with processing of visual inputs by local sub-networks within V1 that are tuned to combinations of sensory features.

## Introduction

The brain first dissects the content of a visual scene into its components, such as oriented edges, and then further combines these to form representations of complex objects. For example, seen through a small aperture (as given by the receptive field size of a neuron), only the motion component perpendicular to an edge can be inferred, while the motion component parallel to the edge remains ambiguous. Because of this “aperture problem,” integration of motion from multiple edges is required to unambiguously resolve the motion direction of an object (Adelson and Movshon, 1982). To study motion integration in visual cortex, plaid pattern stimuli have been extensively used, where two differently oriented gratings are superimposed leading to the percept of a moving plaid (Zeki, 1974; Movshon et al., 1983). In monkeys, the classical view is that cells in primary visual cortex (V1) are selective primarily to the movement of individual oriented components, and are therefore classified as component-selective neurons. In contrast, pattern-selective cells, which respond to the overall motion of a plaid, are found more often in the specialized higher cortical area MT (Movshon et al., 1983; Rodman and Albright, 1989; Stoner and Albright, 1992), explained by feedforward integration of V1 responses within MT (Simoncelli and Heeger, 1998; Rust et al., 2006).

As in many other species, neurons in mouse visual cortex have been found to be highly tuned to the orientation and direction of moving bars or gratings (Mrsic-Flogel et al., 2007; Niell and Stryker, 2008; Andermann et al., 2011; Kreile et al., 2011; Marshel et al., 2011; Roth et al., 2012) but pattern motion integration has not been tested (but see Discussion; Juavinett and Callaway, 2015). We asked whether pattern integration might already occur in V1, since rodents' primary sensory cortices have been shown to possess a wiring scheme that could support integration of individual features. Specifically, recordings in rat cortical slices have revealed the presence of a connectivity scheme across layers that partitions the cortex into neuronal sub-networks, suitable for binding independent streams of sensory information (Kampa et al., 2006).

Here, we used two-photon calcium imaging of neuronal populations in mouse V1 during presentation of moving gratings and plaid patterns to reveal the level of feature integration at this stage of the visual pathway. We used traditionally defined models for classifying component and pattern cells (Movshon et al., 1983) under a novel Bayesian framework that explicitly incorporates trial-to-trial variability in the analysis. We also estimated the degree of neuronal facilitation and suppression when using plaid pattern stimulation and found that a majority of cells were suppressed even though some neurons showed facilitation in their responses to plaid pattern stimulation. Neurons not accounted for by this two-step analysis had responses that suggested a strong influence of local recurrent interactions. Our results indicate that in the mouse a certain degree of pattern integration and generation of pattern selectivity already takes place at the level of the V1. In addition, neuronal responses revealed a footprint of the underlying circuit architecture tuned for sensory integration.

## Materials and methods

### Animal preparation

All animal procedures were carried out according to the guidelines of the University of Zurich, and were approved by the Cantonal Veterinary Office. Seven C57BL/6 mice and one Thy1-YFP mouse with C57BL/6 background (2–4 months old, of either sex) were anesthetized by intraperitoneal injections with 2.7 ml/kg of a solution made of one part fentanyl citrate and fluanisone (Hypnorm; Janssen-Cilag, UK) and one part midazolam (Hypnovel; Roche, Switzerland) in two parts of water. Atropine (0.3 mg/kg) and dexamethasone (2 mg/kg) were administered subcutaneously to reduce secretions and edema. Lactate-Ringer solution (composition in mM: 130.9 Na^+^, 5.4 K^+^, 1.84 Ca^2+^, 111.7 Cl^−^, 28.3 L-Lactat) was regularly injected subcutaneously to prevent dehydration. Pinch reflexes were used to assess the depth of anesthesia. Additional doses of anesthetic were given as needed to maintain anesthesia. Imaging typically lasted between 2 and 4 h, as several planes were acquired in each animal. Recordings from a total of 56 neuronal populations were obtained.

### Two-photon imaging

Two-photon calcium imaging of neuronal responses upon visual stimulation was performed as previously described (Roth et al., 2012). Briefly, V1 was identified using intrinsic imaging. A small craniotomy (between 500 × 500 and 800 × 800 μm^2^) was opened above V1, the dura removed and the exposed cortex superfused with artificial cerebrospinal fluid (ACSF) (135 mM NaCl, 5.4 mM KCl, 5 mM Hepes, 1.8 mM CaCl_2_, 1 mM MgCl_2_, pH 7.2, with NaOH). The calcium indicator Oregon Green BAPTA-1-AM ester (OGB-1; 50 μg; Invitrogen, Basel, Switzerland) was dissolved in 2 μl DMSO plus 20% Pluronic F-127 (BASF, Germany) and diluted with 37 μl standard pipette solution (150 mM NaCl, 2.5 mM KCl, 10 mM Hepes, pH 7.2) yielding a final OGB-1 concentration of about 1 mM. One microliter of Alexa Fluor 594 (2 mM stock solution in distilled water) was added for visualization of the pipette during two-photon guided OGB-1 injection. The dye solution was pressure ejected under visual control through a glass pipette (4–5 MΩ) at a depth between 150 and 250 μm to stain layer 2/3 neurons (Stosiek et al., 2003). Brief application of sulforhodamine 101 (SR101; Invitrogen) to the exposed neocortical surface resulted in co-labeling of the astrocytic network (Nimmerjahn et al., 2004). Following dye injection the craniotomy was filled with agarose (type III-A, Sigma; 1% in ACSF) and covered with an immobilized glass cover slip.

Fluorescence changes were measured using a custom-built two-photon microscope with 100 fs laser pulses at 830 or 870 nm wavelength provided by a Ti:sapphire laser system (Spectra-Physics). We modulated laser intensity using a Pockel's cell (Conoptics). The microscope was equipped with either a 40 × water immersion objective (LUMPlanFl/IR; 0.8 NA; Olympus) or a 20 × water immersion objective (XLUMPlanFI; 0.95 NA; Olympus). 256 × 256 pixel image frames were acquired at 2 Hz using custom written software (LabView; National Instruments, USA). The field of view size varied between 183 × 183 and 357 × 357 μm^2^.

### Visual stimulation

**Figure 1:**
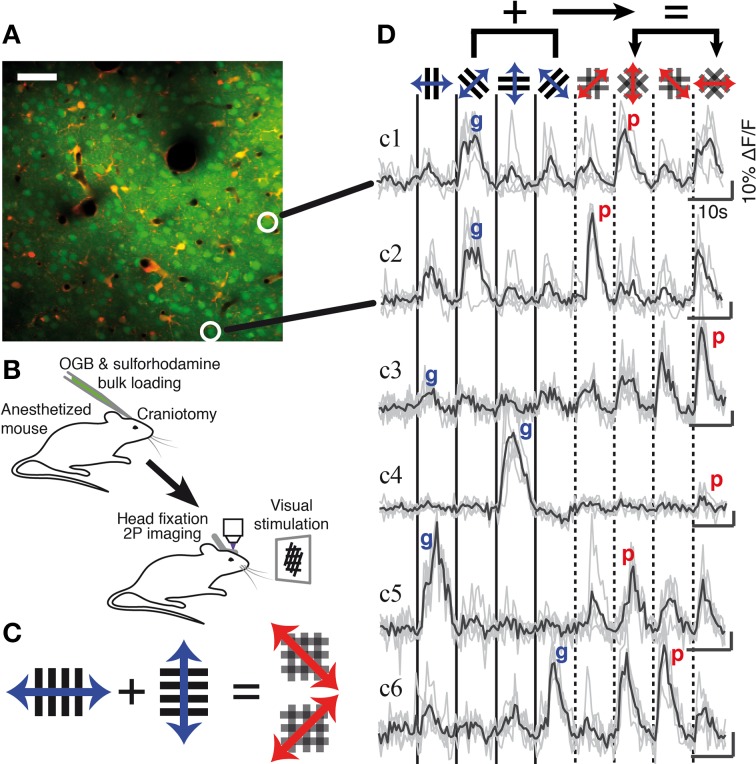
**Two-photon calcium imaging of gratings and plaid pattern evoked responses in mouse V1. (A)** Example two-photon image of layer 2/3 neurons at a depth of 273 μm in V1; neurons were labeled with the calcium indicator OGB-1/AM (green) and astrocytes counterstained with sulforhodamine (SR101, red). The circled neurons correspond to the indicated traces shown in **(D)**. Scale bar: 50 μm. **(B)** Anaesthetized mouse two-photon calcium imaging protocol. **(C)** Visual stimuli. Individual grating components or plaid stimuli composed of orthogonal gratings drift back and forward during a single presentation. The relative drift direction of the grating components defines the direction of the resulting plaid motion. **(D)** Single trial (gray) and average (black) calcium traces recorded in response to grating and plaid stimuli (indicated by icons at top). Each classified response class is represented (see also Figure 2). “g” and “p” labels on each trace indicate the maximum grating and plaid responses for that neuron and were used to calculate a Modulation Index (MI; see Material and Methods). Scale bars: 10 s and 10% ΔF/F.

Visual stimuli were presented on a 21 inch CRT monitor placed 30 cm in front of the contralateral eye roughly at 60° along the body axis of the anesthetized mouse, covering approximately 66 × 77° of the visual field. Full-field, full contrast drifting square wave gratings were generated by the VisionEgg software package (Straw, 2008; see Figure 1C). Square wave gratings of four different orientations (0, 45, 90, and 135°) were presented sequentially, followed by presentation of four plaid stimuli composed of superimpositions of two orthogonal gratings. The contrast of the superimposed gratings was reduced to 50% and their intersection created nodes of 100% contrast. To reduce the total number of visual stimuli, gratings and plaid stimuli drifted alternately in both directions within a single trial (0–180, 45–225, 90–270, and 135–315°). Stimulation time was 2 s per direction leading to a total of 4 s per grating of a single orientation at a temporal frequency of 1.5 Hz and a spatial frequency of 0.04 cycles per degree. Stimulation periods were separated by 5 s of a gray screen. Stimuli were presented until at least five successful trials were collected for each imaged region.

### Analysis of calcium transients

Data were analyzed with ImageJ (National Institute of Mental Health NIH) and MATLAB (Mathworks). Cells were defined manually by drawing regions of interest (ROIs) around cell bodies. Fluorescence signals were averaged from all pixels inside a ROI and calcium signals were expressed as relative fluorescence changes (Δ*F/F*) after subtraction of a background (typically from a blood vessel lumen). Response amplitudes were calculated by averaging three points around the peak fluorescence change (a 1.5 s time window around the peak) for each stimulation epoch. The mean pre-stimulus fluorescence was subtracted from each response. To estimate neuronal responsiveness, only neurons with responses larger than two times the standard deviation of the baseline, which persisted in more than 50% of the trials, were considered as showing significantly evoked activity (see also Roth et al., 2012). To estimate neuronal selectivity, neurons that responded selectively to any stimulus were obtained by performing an ANOVA test between the responses to all stimuli (gratings and plaids) at a *p*-value threshold of 0.05. Neurons that passed this test were dubbed “selective,” and were used for further analysis. We calculated the response reliability for each neuron by measuring the average trial-to-trial correlation of the peak responses for a single neuron, over all stimuli.

### Bayesian model-based analysis framework

**Figure 2:**
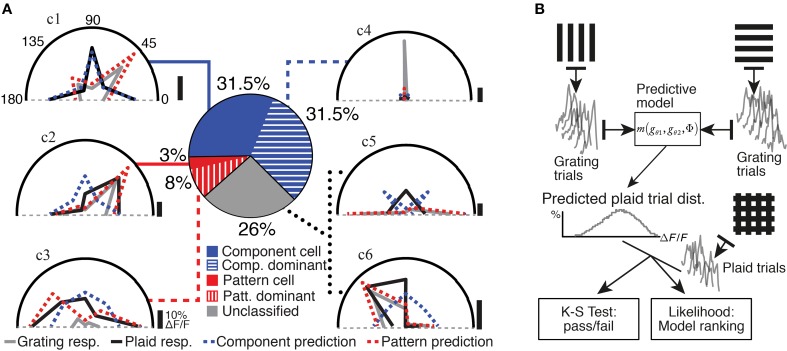
**Analysis and classification of the neuronal responses. (A)** The analysis and classification of the example neurons presented in Figure 1D (traces labeled c1 through c6) are illustrated. Grating (gray) and plaid (black) responses are shown on polar plots of stimulus drift angle (degrees); component and pattern model predictions are also indicated (blue and red dashed curves, respectively); vertical scale bars indicate 10% ΔF/F for each polar plot. Middle: Pie chart shows our quantification of response classes for the entire data set of responsive and selective neurons in mouse V1. **(B)** A schematic diagram of our model-based analysis method (see Material and Methods). The set of single-trial responses to two grating component stimuli and are used to form a prediction of the response of a neuron to a plaid stimulus under an arbitrary predictive model. This prediction combines all possible combinations of trials of the component stimuli, to produce a predicted distribution for single-trial responses. This prediction is then compared with the set of experimentally obtained single-trial responses to plaid stimuli. A Kolmogorov–Smirnov test with multiple-comparisons correction provides a pass/fail value for each model, for each neuron. The likelihood of observing the experimentally obtained single-trial plaid responses under each model prediction is also estimated, in order to rank a set of models. This framework therefore provides several distinct benefits: the ability to reject or accept a model as providing a useful prediction, in a rigorous statistical sense; the ability to rank any number of models in a statistically justified manner; and the ability to determine whether or not the predictions between two accepted models are significantly different, within a rigorous statistical framework.

To classify the responses of neurons we used a novel Bayesian model-based analysis framework, applied to an extension of the Partial Correlation (PC) method (Movshon et al., 1983; Rodman and Albright, 1989; Movshon and Newsome, 1996; Baron et al., 2007). Here we describe our formal statistical framework for rejecting a model, as well as for ranking the predictions for several models. A schematic figure of the framework is shown in Figure 2B. The set of observed grating responses for a neuron is denoted *G*, with single-trial responses from the set denoted as *g*_θ*j, i*_. Here θ*j* is a single stimulus orientation and *i* is a trial index ranging between 1 and the number *T* of single-trial observations for the given stimulus. For convenience, we also define the vector $g\overset{\text{def}}{=}\left\{\bar{g_{\theta 1}},\;\bar{g_{\theta 2}},\;\ldots \right\}$, consisting of all trial-averaged responses over the set of different grating stimuli. Here $\bar{g_{\theta j}}$ denotes the trial-averaged response for a single grating stimulus θ*j*, that is $\bar{g_{\theta j}}=1/T\sum {}_{i}g_{\theta j,i}$.

By analogy we construct the set *P* of all plaid responses for the neuron, with single-trial responses denoted *p*_θ1+θ2, *i*_ (with θ1 and θ2 defining the two grating components that comprise the plaid stimulus); and define the vector $p\overset{\text{def}}{=}\left\{\bar{p_{\theta 1+\theta 2}},\;\bar{p_{\theta 1+\theta 3}},\;\ldots \right\}$ containing trial-averaged responses to the set of plaid stimuli.

Single-trial responses to one or more grating stimuli (denoted *g*_θ*j, h*_, *g*_θ*k, i*_, … ) were used to form a prediction for a single-trial response of the same neuron to a plaid stimulus ${\hat{p}}_{\theta 1+\theta 2}$, under an arbitrary and possibly non-linear model ${\hat{p}}_{\theta 1+\theta 2,l}\;=\;m\left(g_{\theta j,h},\;g_{\theta k,i},\;\ldots ,\;\phi \right)$. Model *m* is arbitrary, and ϕ is an arbitrary set of model parameters. The precise models used in this work are described below.

The model *m* is assumed to be commutative over a given set of grating component inputs, such that *m*(*g*_θ1, *h*_, *g*_θ2, *i*_, ϕ) = *m*(*g*_θ2, *i*_, *g*_θ1, *h*_, ϕ). All combinations of appropriate single-trial responses are used to generate a set ${\hat{P}}_{m}$ of predicted plaid responses for the neuron. The predicted responses ${\hat{P}}_{m}$ are compared with the set *P* of observed single-trial responses to plaid stimuli. Firstly, a Kolmogorov–Smirnov (K–S) test is used to compare the predictions from a given model to the set of single-trial responses to an individual plaid stimulus, resulting in a K–S test result for each plaid stimulus. These test results are combined using a Holm–Bonferroni correction for multiple comparisons, to accept or reject the predictions from a given model under an α = 5% statistical significance threshold.

Subsequently, the likelihood of observing the set of experimental plaid responses under the model *m* is estimated to perform model ranking; this likelihood is given by $Pr\left(X=P|m,\phi \right)\overset{\text{def}}{=}\prod {}_{\theta 1,\theta 2,i}Pr\left(x_{\theta 1+\theta 2}=p_{\theta 1+\theta 2,i}|m,\phi \right)$, by combining the likelihoods of each of the single-trial responses to the set of plaid stimuli. The individual plaid response probability distributions *Pr*(*x*_θ1+θ2_|*m*, ϕ) are estimated by direct sampling from the single-trial grating response distributions approximated by Normal distributions, such that *Pr*(*x*_θ*j*_)~*Norm*(μ_θ*j*_, σ_θ*j*_). Here μ_θ*j*_ and σ_θ*j*_ are the mean and standard deviations over the single-trial responses *g*_θ*j, i*_ to the grating stimulus θ*j*. A set of samples *M*_θ1+θ2_ from *Pr*(*x*_θ1+θ2_|*m*, ϕ) is generated by Monte-Carlo sampling of *m*(*y*_θ*j*_, *y*_θ*k*_, …, ϕ), where *y*_θ*j*_~*Pr*(*x*_θ*j*_) and so on. The likelihoods for single-trial observed plaid responses *Pr*(*x*_θ1+θ2_ = *p*_θ1+θ2, *i*_|*m*, ϕ) are then estimated via a Kernel density method over *M*_θ1+θ2_.

Likelihoods are then used to rank relative accuracy over several models; a difference of more than 5 decibans was considered significant evidence in favor of one model over another (Jeffreys, 1961). We note that our value for *Pr*(*X* = *P*|*m*, ϕ) is not a true likelihood due to the difficulty in estimating the true probability of a given observation under a continuous probability distribution. However, any set of likelihoods being compared are always estimated for the same neuron over the same set of experimental responses, from distributions with identical variance.

### Plaid response models

Our models for component and pattern responses used in this paper are extensions of those used in the partial correlation framework (Movshon et al., 1983; Rodman and Albright, 1989; Movshon and Newsome, 1996; Baron et al., 2007). For simplicity of notation we will leave off the trial index; that is, *g*_θ1_ will refer to a single-trial response *g*_θ1, *i*_ from an arbitrary trial *i*, under presentation of stimulus θ1. Our “component model” assumes that a predicted single-trial response ${\hat{p}}_{\theta 1+\theta 2}$ to a plaid stimulus is given by the sum of two single-trial responses to the two individual grating components, normalized by a factor *k*, such that ${\hat{p}}_{\theta 1+\theta 2}=m\left(g_{\theta 1},\;g_{\theta 2},\;k\right)=k\left(\frac{g_{\theta 1}+g_{\theta 2}}{2}\right)$. This model encompasses the original “component cell” proposal of Movshon (by constraining *k* = 2); a prediction by the mean of the two grating components (by constraining *k* = 1); and any other degree of suppression or facilitation by allowing *k* to adopt a value that optimally predicts the response for a single cell. Our “pattern model” assumes that a response ${\hat{p}}_{\theta 1+\theta 2}$ to a plaid stimulus is predicted by the grating component *g*_θ3_ that drifts in the same direction as the combined plaid, normalized by a factor *k*: ${\hat{p}}_{\theta 1+\theta 2}=m\left(g_{\theta 3},\;k\right)\;=\;k\cdot g_{\theta 3}$. In our model-based analysis, *k* is permitted to adopt the optimal value for each cell that best explains the average response of that cell.

Our framework is modular, and any alternative model that predicts a set of target responses from a set of observed responses can be included. Since our framework provides a method for ranking several models via response likelihood, any number of response models can be used if desired.

### Partial correlation analysis

For comparison with our Bayesian model-based analysis framework, we compared our technique against the PC approach used in previous literature (Movshon et al., 1983; Rodman and Albright, 1989; Movshon and Newsome, 1996; Baron et al., 2007). Briefly, predicted trial-average responses under “pattern cell” and “component cell” models were formed. Pattern cells were defined such that the response to a given plaid stimulus was identical to the grating response for the grating drifting in the same directions as the vector sum of plaid component drift directions. Component cells were defined such that responses to a given plaid stimulus were the linear sum of the grating responses to the two plaid components. These idealized pattern and component cell responses to the plaid stimulation were identical to the ones used in our analysis framework, however, as stated above, the model-based analysis also encompasses the variability of neuronal responses, while the PC uses only average responses.

For a given neuron, the recorded trial-averaged plaid responses are denoted ***p***; the predicted plaid responses under the pattern and component cell models are denoted ${\hat{p}}_{p}$ and ${\hat{p}}_{c}$, respectively. The correlations between observed and predicted responses are then calculated by $\rho_{c}=corr\left(p,{\hat{p}}_{c}\right)$ and $\rho_{p}=corr\left(p,{\hat{p}}_{p}\right)$, and the inter-prediction correlation is given by $\rho_{pc}=corr\left({\hat{p}}_{c},{\hat{p}}_{p}\right)$. The partial correlation measures *R*_*c*_ and *R*_*p*_ are then given by $R_{c}=\left(\rho_{c}-\rho_{p}\rho_{pc}\right)/\sqrt{\left(1-\rho_{p}^{2}\right)\left(1-\rho_{pc}^{2}\right)}$ and $R_{p}=\left(\rho_{p}-\rho_{c}\rho_{pc}\right)/\sqrt{\left(1-\rho_{c}^{2}\right)\left(1-\rho_{pc}^{2}\right)}$; the *Z*-scored versions of these measures are given by $Z_{c}=\frac{1}{2}\left(\log_{10}\frac{1+R_{c}}{1-R_{c}}\right)/\sqrt{1/d}$ and $Z_{p}=\frac{1}{2}\left(\log_{10}\frac{1+R_{p}}{1-R_{p}}\right)/\sqrt{1/d}$, where *d* is the degrees of freedom (number of stimuli minus 3; here *d* = 5). For a cell to be classified as *component*, *R*_*c*_ must be greater than *R*_*p*_ and also greater than zero, in a one-tailed Z test at a significance above chance level of α = 0.1 (Movshon et al., 1983; Gizzi et al., 1990; Scannell et al., 1996; Baron et al., 2007). The same test is used to classify responses as *pattern* cells. The remaining cells that meet neither criterion are left unclassified.

### Modulation index (MI)

We defined an index that estimates the degree of suppression or facilitation involved in the response to plaid stimuli, compared with the response of the same neuron to grating stimuli. The modulation index (MI) is given by the formula $MI=\frac{\max\left(p_{i}\right)-\max\left(g_{i}\right)}{\max\left(p_{i}\right)+\max\left(g_{i}\right)}$ where max(***g***_*i*_) and max(***p***_*i*_) are the maximum trial-averaged responses of a neuron *i* over the set of grating and plaid stimuli, respectively.

### Response classification procedure

Our model-based analysis framework was applied to the responses of selective neurons (see above), that responded to at least one grating and at least one plaid stimulus. For each neuron, we obtained a pass/fail decision and likelihood estimate for each of the pattern and component models, and measured the MI (see above). Neurons for which only a single model was accepted were classified into the corresponding category (*component-classified* or *pattern-classified*). When both models were accepted for a given neuron, the model likelihoods were used to rank the models (see above). If one model performed significantly better for a given neuron, that neuron was classified into the corresponding category (*component-classified* or *pattern-classified*).

Neurons for which both models performed equally, or where both models were rejected, were subjected to a modulation test. If the MI for these neurons was >0.33 or < -0.33 then the neuron was categorized *pattern-dominant* or *component-dominant*, indicating a very strong preference for one stimulus class. These categories also included neurons that responded only to grating or only to plaid stimuli.

### Grating and plaid selectivity index

We defined a selectivity index (SI) that can be applied identically to both grating and plaid stimuli. The SI for a neuron is defined over a set of stimulus responses ***s***, where ***s*** is a vector of trial-averaged responses over a set of stimuli: $s\overset{\text{def}}{=}\;\left\{\bar{s_{1}},\;\bar{s_{2}},\;\ldots ,\;\bar{s_{N}}\right\}$, with any negative responses clipped to zero. The index is calculated with $SI\;\overset{\text{def}}{=}\;1-\left[-1+\sum {}_{j}\bar{s_{j}}/\max\left(s\right)\right]/\left(N-1\right)$. Neurons that are highly selective over a set of stimuli (for example, neurons that are highly orientation tuned in response to a set of drifting gratings of varying orientation) have an SI close to 1. Neurons that are broadly tuned or unselective for a set of stimuli (for example if a neuron responds identically to each stimulus in a set) have an SI close to zero. This metric can be computed separately over the set of grating or plaid stimuli, resulting in independent estimates of selectivity over grating and plaid responses.

### Grating/plaid response pairwise similarity analysis

To quantify the relationship between grating and plaid responses across the recorded population, in a non-parametric manner, we examined pairs of neurons to measure the similarity between their grating and plaid responses. Similarity metrics *r*_*g*_(*i, j*) and *r*_*p*_(*i, j*), based on Pearson correlation coefficients, were calculated between the vectors of trial-averaged responses for two neurons *i* and *j*, separately for the set of grating and the set of plaid responses. For example, if the vector of trial-averaged grating responses for a neuron *i* is denoted ***g***_*i*_ as above, then *r*_*g*_(*i, j*) = *corr*(***g***_*i*_, ***g***_*j*_), where $corr\;\left(x,y\right)\;\overset{\text{def}}{=}\;\frac{\sum \left(x_{i}-\bar{x}\right)\;\sum \left(y_{j}-\bar{y}\right)}{\sqrt{\sum {\left(x_{i}-\bar{x}\right)}^{2}}\;\sqrt{\sum {\left(y_{i}-\bar{y}\right)}^{2}}}$ is the Pearson correlation coefficient between two vectors. We define a plaid similarity metric *r*_*p*_(*i, j*) = *corr*(***p***_*i*_, ***p***_*j*_) analogously to *r*_*g*_(*i, j*). These similarity metrics permit an estimate of the predictability of plaid responses from grating responses in a non-parametric manner, by comparing the relationship between *r*_*g*_ and *r*_*p*_ for the same pairs of neurons.

### Analysis of grating and plaid similarity indices for unclassified neurons

We estimated the number of simple rules that are required to reproduce the distribution of plaid and grating similarity indices *r*_*g*_ and *r*_*p*_ for the unclassified neurons (**Figure 5B**). We assumed that two neurons obeying the same rule will have consistent similarity for both grating and plaid responses (i.e., *r*_*g*_ = *r*_*p*_ for these neurons). We randomly divided the unclassified neurons into *n* groups of equal size, and set the plaid similarity indices *r*_*p*_ to be equal to *r*_*g*_ within each group. This procedure implies that pair-wise similarity indices within groups were aligned, whereas similarity indices between groups were left unchanged from our experimental observations. We performed this resampling 100 times for each number of rules *n* [*n* = (1.10)], and collected the resulting distributions of pair-wise correlations. A two-dimensional, two-tailed Kolmogorov–Smirnov test was used to compare the resampled distributions with the experimental distribution shown in **Figure 5B**. If the distributions were significantly different for a given number of rules *n* (*p* < 0.05), then we considered that number of rules to be insufficient to explain the experimental distribution of pair-wise correlations (i.e., reject the null hypothesis).

## Results

### Two-photon calcium imaging of grating and plaid pattern responses

We measured visually evoked neuronal responses in layer 2/3 of V1 in anesthetized mice using two-photon calcium imaging with Oregon Green BAPTA-1 (OGB-1) (Figures 1A,B; *n* = 8 mice; 56 imaged regions containing 4088 neurons in total). Four drifting high-contrast gratings and four plaid stimuli composed of orthogonal gratings were presented to the contralateral eye of the animal (Figure 1C). Of all imaged neurons 42% were responsive; 51% of the responsive population were selective for at least one of the visual stimuli and were considered for further analysis (*n* = 877 neurons; selectivity defined by ANOVA at *p* < 0.05; see Materials and Methods). Neurons displayed a variety of responses, with a majority of cells responding strongly for both gratings and plaid pattern stimulation (example neurons c1, c2, c5, and c6 in Figure 1D) while other cells responded only to either gratings (neuron c4) or plaid patterns (neuron c3). Notably, cells that responded similarly to the set of grating stimuli could respond very differently to the set of plaid stimuli, and vice versa (e.g., neuron c1 compared to neuron c2; see also **Figure 5**). Neurons in mouse V1 presented a wide diversity of responses to gratings and plaid pattern stimulation. Therefore, we first functionally classified these diverse neuronal responses as *component*- or *pattern-classified*.

### Classification of component and pattern responses

Two classes of motion-integrating response classes for plaid stimuli have previously been described for neuronal responses in visual cortex (Movshon et al., 1983). *Pattern-classified* neurons are defined as neurons preferring a plaid stimulus that drifts in a direction identical to the preferred motion direction tested with grating stimuli. In contrast, *component-classified* neurons respond best to plaid stimuli that contain a grating component matching the preferred grating drift direction for that neuron. We assigned neurons to these two response classes using an extension of the Partial Correlations analysis framework (Movshon et al., 1983; Rodman and Albright, 1989; Movshon and Newsome, 1996; Baron et al., 2007), which predicts the response of a neuron to the set of plaid stimuli based on its responses to the set of grating stimuli (see Materials and Methods; Figure 2B). The predictions of two models, corresponding to *pattern* and *component* response classes (see Materials and Methods), were compared to the actual plaid pattern responses (see Figure 2; dotted lines in polar plots indicate predictions for the example cells shown in Figure 1D). The performance of both models was evaluated statistically to accept or reject their predictions as well as to provide a ranking of the two models (see Materials and Methods; Figure 2B). If experimentally recorded responses were significantly more likely under one model than the other, the neuron was categorized accordingly as either *component-classified* or *pattern-classified* (Figure 2). In our analyzed set of responsive and selective neurons 31.5% were *component-classified* (275/877 neurons) and 3% were *pattern-classified* (29/877 neurons).

Some of the neurons that did not fall clearly into the component- or pattern-classified categories showed clear preferences for either only gratings or only plaid stimuli (see for example neurons c3 and c4; Figures 1D, 2). This selectivity was best captured by comparing the strongest response of a neuron to a grating stimulus (labeled “g” in Figure 1D) with the strongest response to a plaid pattern (labeled “p” in Figure 1D). Using a modulation index (see Materials and Methods) we defined two further classes: Neurons with strongly suppressive (MI < -0.33) or facilitatory responses (MI > 0.33) were placed into *component-dominant* or *pattern-dominant* categories, respectively (Figure 2). We found 31.5% *component-dominant* neurons (275/877) and 8% *pattern-dominant* cells (70/877) among the analyzed neuronal population.

In summary, our classification procedure accounted for more than 70% of the responsive and selective neurons in mouse V1. *Component-selective* cells comprised 63%, divided equally between *component-classified* and *component-dominant* neurons. In contrast, 11% of neurons showed *pattern-selective* responses with 3% being *pattern-classified* and the remainder being *pattern-dominant* neurons (Figure 2). The remaining 26% of unclassified responses showed more complex responses discussed further below. We conclude that, in addition to the typical *component-selective* responses observed in V1 of several species (Movshon et al., 1983; Gizzi et al., 1990; Baron et al., 2007), a small but significant proportion of neurons selective for pattern stimuli also exist in mouse V1.

### Bayesian model-based classification outperforms partial correlation analysis

**Figure 3:**
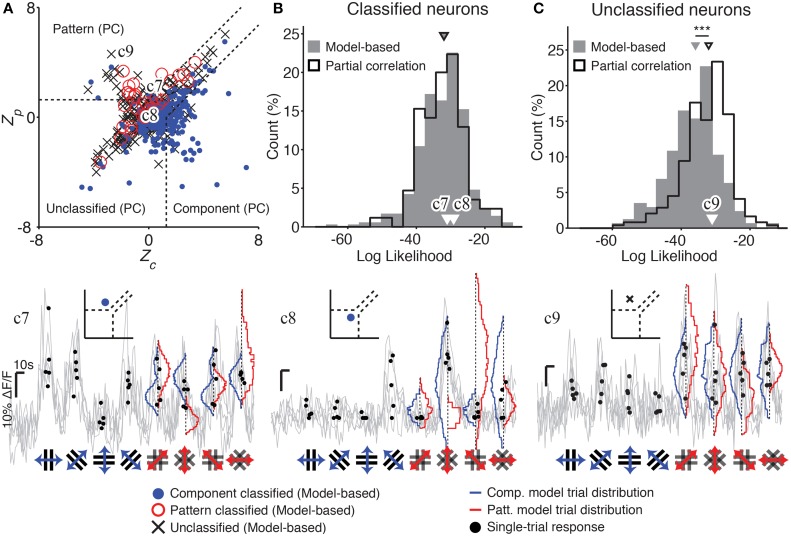
**Model-based classification performs better than partial correlation (PC) analysis. (A)** The distribution of Z-scored partial correlations for the component (*Z*_*c*_) and pattern (*Z*_*p*_) models is plotted, along with the decision boundaries used to classify responses under the PC analysis (dashed lines; see Materials and Methods). Each cell is plotted with a color and symbol according to its classification under our model-based analysis method (see text; Materials and Methods). Many of the neurons left unclassified by the PC analysis are successfully classified by our model-based approach [see cells plotted in the “Unclassified (PC)” region]. In addition, some cells classified by the PC analysis are classified differently, or left unclassified, by our model-based approach. **(B)** Cells classified by our model-based approach (gray) are just as well-predicted as cells classified by PC analysis (black; median LL −32 vs. −32, *p* = 0.88, rank-sum test, *n*_MB_ = 355, *n*_PC_ = 85). **(C)** However, cells left unclassified by the PC analysis could have been reasonably predicted by one of the two models (black; high LL). In contrast, cells left unclassified by our model-based approach were much more poorly predicted by one of the two models (gray; lower LL); this difference between the two classification approaches was statistically significant (median LL -36 vs. -32, *p* < 0.001, rank-sum test, *n*_MB_ = 177, *n*_PC_ = 244). Example traces c7–c9 illustrate cells which are differently classified by PC and our model-based approach. Gray traces indicate single-trial calcium responses; the single-trial responses used in our analysis are indicated as black dots. Blue and red curves indicate predicted single-trial distributions under the component and pattern models, respectively, generated as part of our model-based analysis. Insets in c7–c9 indicate both the model-based and PC classification of the corresponding cell. Labels in **(A–C)** indicate the measures corresponding to each example cell. Trace c7 shows a cell which is classified as a “pattern” cell by PC analysis, but “component” cell by our model-based analysis. The observed single-trial responses to plaid stimuli (black dots) are more likely under the component model than the pattern model (blue vs. red curves). Trace c8 shows a cell that could not be classified under PC analysis, but was classified as a component cell by our model-based analysis. Trace c9 shows a cell that was classified as a pattern cell by PC analysis, but was left unclassified by our analysis since the single-trial responses to plaid stimuli were equally well predicted by the component and pattern models (blue vs. red curves). Scale bars: 10 s and 10% ΔF/F. ^***^p < 0.001.

Our model-based analysis method offers several advantages over classification of pattern and component cells using PC analysis. Firstly, we are able to classify a greater proportion of responses into pattern and component classes (304 vs. 85 cells; Figure 3A), without loss of performance (no difference in log likelihoods; Figure 3B). Model predictions were also significantly better correlated with the recorded responses of successfully classified neurons than with responses of unclassified neurons (Supplementary Figure 1; median correlations 0.55 vs. 0.08; *p* < 0.01, rank-sum test). For some cells, the trial-averaged response used by PC analysis was a poor description of the cell's full response, so that our model-based approach assigned a different category than PC analysis (e.g., Figure 3 trace c7). The cells left unclassified by our model-based method had responses that were poorly explained by either of the pattern or component models (low log likelihoods; Figure 3C), or were equally well explained by both models (see Figure 3 trace c9). However, cells left unclassified under the PC analysis in general had responses that fit reasonably well into one of the pattern or component models (significantly higher log likelihoods, *p* < 0.001; Figure 3C; see Figure 1 trace c2 and Figure 3 trace c8).

### Pattern integration is more facilitatory than component integration

In primate area MT pattern integration is thought to rely on convergent projections onto MT neurons. Plaid patterns composed of overlaid gratings may be integrated by combining inputs in a supralinear manner (Rust et al., 2006) resulting in facilitated neuronal responses during plaid pattern stimulation compared with grating stimulation. We could not directly measure supralinear integration since this would involve recording the sub-threshold membrane potential. However, we used the spiking output of the neurons to analyze the extent of facilitation during pattern integration by comparing grating and plaid responses using the MI for all responsive and selective neurons. Since the calcium dye OGB-1 has a complex non-linear and saturating response, we cannot determine whether summation from grating components to plaid responses is supra- or sub-linear (Nauhaus et al., 2012). However, we can report whether responses to grating or plaid stimuli were stronger in general. We note that our approach will tend to underestimate the extent of plaid facilitation in V1.

**Figure 4:**
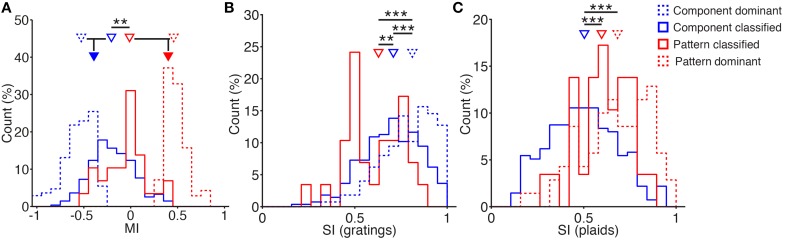
**Differences in modulation and tuning between component and pattern cells. (A)** Modulation Index (MI) distributions of *component-classified* (blue), *pattern-classified* (red), *component-dominant* (blue dashed), and *pattern-dominant* (red dashed) cells (percentages of indicated classes). Triangles indicate the medians of the distributions, with grouped medians indicated below. *Pattern-classified* cells have a significantly higher modulation index than *component-classified cells*, highlighting more facilitation in this group (medians 0.0 vs. −0.2, *p* < 0.01, rank-sum test). Grouping cells according to their overall component- and pattern-preference (indicated by filled triangles) highlights the considerable facilitation of *pattern-selective* responses (medians −0.4 vs. 0.4, respectively). **(B)** Grating selectivity index (SI) distribution of classified component (blue) and pattern (red) cells (see Material and Methods; percentages are of indicated classes). *Pattern-classified* cells are significantly less selective for grating stimuli (median SI_*g*_ 0.6 vs. 0.7, *p* < 0.01, rank-sum test, *n*_C_ = 275 *n*_P_ = 29). *Component-dominant* cells are highly selective over grating stimuli (median SI_g_ 0.8, *n*_CD_ = 275). **(C)** Plaid SI distribution of *component-classified* (blue) and *pattern-classified* (red) cells (see Material and Methods; percentages are of indicated classes). *Pattern-classified* cells are significantly more selective for plaid stimuli (median SI_p_ 0.6 vs. 0.5, *p* < 0.001, rank-sum test, *n*_C_ = 275 *n*_P_ = 29). *Pattern-dominant* cells are highly selective over plaid stimuli (median SI_p_ 0.7, *n*_PD_ = 70). ^**^p < 0.01; ^***^p < 0.001.

Overall, most neurons in the analyzed population expressed weaker responses to plaid stimulation compared with grating stimulation (median MI: −0.3) consistent with previous reports for V1 (Morrone et al., 1982). However, almost a quarter of these showed facilitation (24%; MI > 0). Interestingly, when separating the cells into *pattern*- and *component-classified* (as defined by the models), we found that *pattern-classified* cells show more facilitatory integration compared with *component-classified* cells: 40% of the *pattern-classified* neurons were facilitated during plaid pattern stimulation compared with only 20% of the *component-classified* neurons (Figure 4A; median MI: 0.0 vs. -0.2, respectively; rank-sum test: *p* < 0.01). When all *pattern-selective* cells were taken together (*pattern-classified* plus *pattern-dominant*), the median MI was 0.4 compared with -0.4 for all *component-selective* cells (Figure 4A). These findings reinforce the view that component cells code for the orientation of components and undergo cross-orientation suppression during plaid stimulation (Morrone et al., 1982), whereas pattern cells integrate component inputs in a supralinear manner (Rust et al., 2006).

### Pattern and component neurons are differentially tuned over their chosen stimulus types

In order to detect the motion of the plaid stimuli, pattern cells must integrate inputs tuned over a broad range of orientations (see also Figure 6). Hence, if pattern integration occurs locally, the tuning of pattern cells to individual gratings should also be broader. We tested this hypothesis by measuring a tuning SI for the classified neurons over the set of grating stimuli (Figure 4B; see Methods). As expected, we found that *pattern-classified* neurons are significantly more broadly tuned to the orientation of grating stimuli than *component-classified* neurons (Figure 4B; median SI_g_ of 0.6 vs. 0.7; *p* < 0.01, rank-sum test) as previously suggested without being quantified for primate area MT (Rust et al., 2006). *Pattern-classified* cells integrate inputs from the individual components of the presented plaid patterns, resulting in broader orientation tuning when tested with individual drifting gratings.

**Figure 5:**
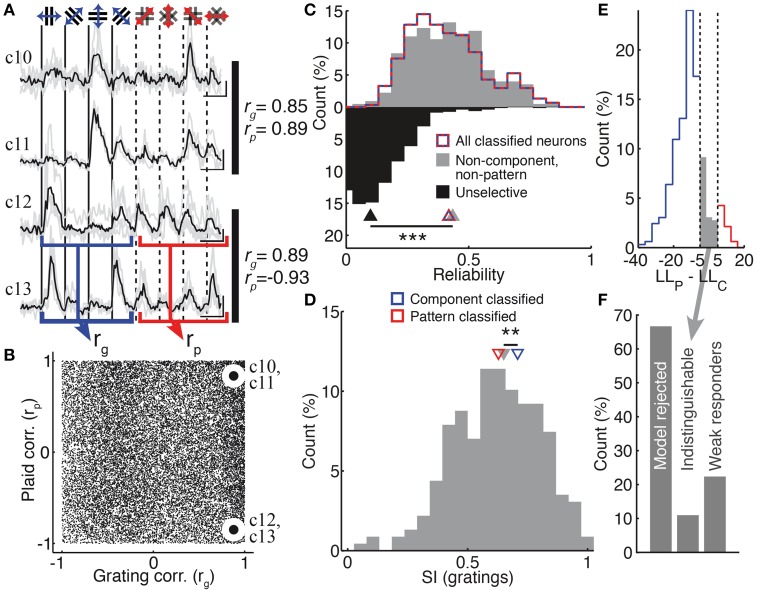
**Non-component, non-pattern neurons have strongly-tuned, reliable and unpredictable responses. (A)** Example response traces of unclassified neurons (see also Figure 1D traces c5 and c6). The responses to grating stimuli were used to compute a pair-wise grating similarity index *r*_*g*_; pair-wise plaid similarities *r*_*p*_ were computed in the same way (values indicated to the right of each pair of traces; see Materials and Methods). Scale bars: 20% ΔF/F, 10 s. **(B)** The population distribution of pair-wise similarities. Indicated points correspond to the pairs of neurons in **(A)**. **(C)** Non-component, non-pattern neurons (gray) were just as reliable as classified neurons (red-blue dashed curve; medians 0.43 vs. 0.41, *p* = 0.58, rank-sum test, *n*_UC_ = 228, *n*_C_ = 304); unselective neurons (black) that were excluded from analysis were significantly less reliable than non-component, non-pattern neurons (medians 0.43 vs. 0.10, *p* < 0.001, rank-sum test, *n*_UC_ = 228, *n*_US_ = 842). **(D)** Non-component, non-pattern neurons were not more broadly tuned than *pattern-classified* neurons, but were more broadly tuned than *component-classified* neurons (medians 0.63, 0.65, 0.71; *p* < 0.01, rank-sum test, *n*_P_ = 29, *n*_C_ = 275, *n*_UC_ = 228) **(E)** The distribution of log-likelihood differences used to assign neurons to pattern (red) and component (blue) classes (percentage of neurons accepted by both models). Discrimination boundaries are shown as dashed vertical lines (see Material and Methods). Some neurons were left unclassified because they fell between these boundaries (gray). **(F)** The proportion of neurons left unclassified either because they were rejected by both models (67%; left bar) or because they fell between the model classification boundaries [11%; middle bar; also indicated in **(E)**]. Neurons that fell between the classification boundaries are a small minority of unclassified neurons. The remaining 22% of unclassified neurons responded to only one stimulus set, but too weakly to be classified as either component- or pattern-dominant (“weak responders”). ^**^p < 0.01.

**Figure 6:**
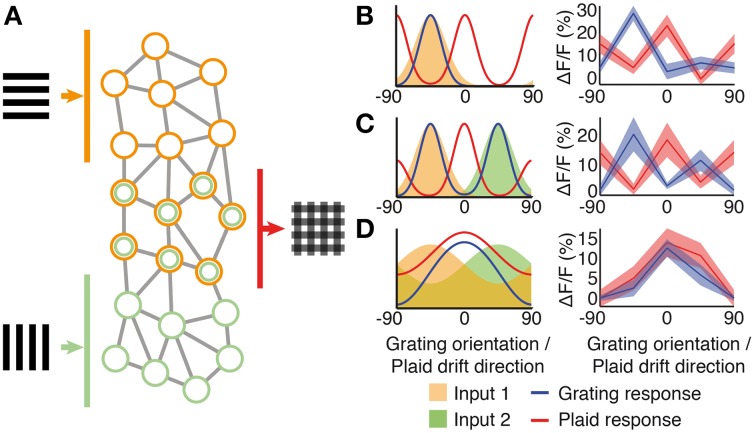
**Pattern integration and hypothesized sub-network circuitry. (A)** Schematic network diagram showing excitatory sub-networks integrating information from two grating components. **(B–D)** Simple integration of tuned input components (shaded areas) can produce a wide range of responses to both gratings (solid curves) and plaids (dashed curves). **(B)** A classical component cell produced by a single narrow-bandwidth orientation- or direction- tuned input (orange shading). Direction-tuned input and responses are shown over 180° of orientation, to match our experimental paradigm. Shown to the right is a neuron with this class of response (see also trace I in Figure 1D). **(C)** An example cell that integrates two narrow-bandwidth input components tuned to two different directions (orange and green shading). Shown to the right is a neuron with this class of response. **(D)** Integration of broadly tuned input components (orange and green shading), produces a broadly tuned “pattern” cell response to drifting gratings (blue curve) and plaid (red curve); shown to the right is a neuron with this class of response (see also pattern classified cell c2 in Figure 1D).

In contrast, *component-classified* neurons were significantly less selective for plaid stimuli than *pattern-classified* neurons (Figure 4C; median SI_p_ 0.6 vs. 0.5; *p* < 0.001, rank-sum test). This is unsurprising, since component cells are expected to respond to two plaid stimuli, both of which contain the preferred component. The selectivity of c*omponent-dominant* and *pattern-dominant* cells was measured over their preferred stimulus class (Figures 4B,C). In both cases, the *-dominant* cells were at least as selective as the corresponding -*classified* cell class.

### Non-pattern, non-component neurons have strongly-tuned and reliable, yet unpredictable, responses

A substantial fraction of neurons (26%) showed significant and selective responses to both gratings and plaid patterns, which, however could not be predicted by either of the pattern or component models (Figure 5A). These neurons showed responses that were as reliable as those of classified neurons (Figure 5C; median trial-to-trial correlations of classified and unclassified neurons: 0.42 vs. 0.43; *p* = 0.6, rank-sum test) and significantly more reliable than unselective neurons that were rejected for further analysis (Figure 5C; median trial-to-trial correlations: 0.10; *p* < 0.001, rank-sum test). They were as sharply tuned as *pattern-classified* neurons but slightly more broadly tuned than *component-classified* neurons (Figure 5D see also Figure 4B; median SI_g_ 0.65 vs. 0.71; *p* < 0.01, rank-sum test). Thus, they were not missed by the classification due to unreliable or broadly tuned responses. Some neurons fell into this category because their responses fell in between predictions from the *component* and *pattern* models (Figures 5E,F; see Materials and Methods). However, neurons with these ambiguous responses comprised a minority of all unclassified neurons—more than six times as many neurons were left unclassified because both models were rejected under our statistical framework (Figure 5F).

We examined the responses of these unclassified neurons to investigate whether a simple alternative model would be able to predict the relationship between their grating and plaid responses. If there would exist a single rule, no matter how complex, that can predict the plaid responses for a neuron given only the grating responses of that neuron, then neurons with similar grating responses must have similar plaid responses. We evaluated whether this condition held for the set of unclassified neurons, by measuring the pair-wise similarity between recorded neurons over the set of their grating and plaid responses (Figures 5A,B, *r*_*g*_ and *r*_*p*_; see Materials and Methods). We found a large variation in relationship between the neurons' pair-wise similarity of grating responses and their similarity of plaid responses. First, we observed a broad range of plaid similarities from pairs of neurons responding similarly to gratings (Figure 5B; high *r*_*g*_, x-axis) to those presenting different responses (Figure 5B; negative *r*_*g*_, x-axis). This was also true when considering their responses to the plaid stimulation (*r*_*p*_, y-axis). Second, we found that the ability to consistently predict plaid from grating responses was essentially nil. Pairs of neurons could be found that showed strong similarity in both grating and plaid responses (Figures 5A,B, neurons c10 and c11), but more often there was no relationship or even an anti-correlated relationship (Figures 5A,B, neurons c12 and c13).

No single model, operating on a single-neuron level to predict plaid responses from responses to gratings, can explain the responses of the unclassified neurons. We therefore considered whether several simple rules taken together could explain the responses of the unclassified population. For this, we performed bootstrap resampling of the pair-wise grating and plaid correlations, under the assumption that a number of arbitrary simple rules exist (see Materials and Methods). We found that at least five such rules, existing concurrently in the cortical population, would be required to explain the wide variation of pair-wise correlations shown in Figure 5B (*p* < 0.05).

The population of unclassified neurons displayed strong, selective, tuned, and reliable responses to the set of grating and plaid stimuli, yet these could not be explained by simple rules relating grating and plaid responses.

## Discussion

We characterized neuronal responses to drifting grating and plaid stimuli in mouse V1 using *in vivo* two-photon calcium imaging, to assess the degree of visual pattern integration in this area. We found a broad spectrum of neuronal responses ranging from neurons responding only to gratings to neurons with complex grating and plaid responses and also neurons that were only activated by presentations of visual patterns.

Our Bayesian model-based analysis classified a greater proportion of responses into pattern and component classes, compared with traditional partial-correlation (PC) analysis, without a reduction in quality of classification (Figure 3). Our model-based classification method uses single-trial responses to form predictions, rather than relying only on averaged responses. This implies that a successful model must not only explain the mean response to a stimulus but also match the distribution of single-trial responses. When the classification under PC analysis differed from our model-based classification, it was often the case that the trial-averaged responses used by PC analysis were not a good description of the full distribution of single-trial responses.

Finally, our Bayesian statistical framework provides the option to reject models entirely, as well as to distinguish between any number of models. In comparison, the PC analysis framework only permits a decision between two models, or to leave the cell unclassified. As a consequence, PC analysis cannot distinguish between a cell that responds weakly or unreliably; a cell that responds strongly but is a mixture between pattern and component models; and a cell with strong and distinct responses that does not match either model.

These observations suggest that our approach is more sensitive and provides a higher-quality classification than PC analysis.

### Control of statistical errors

As part of our classification framework, the set of single-trial model response predictions are tested against the observed responses, using a Kolmogorov–Smirnov test; multiple comparisons correction (MCC; Holm-Bonferroni) is used over the set of predicted stimuli. Models that are not rejected under MCC are then considered for further classification for that cell.

MCC is required to control type-I errors, by limiting either false discovery rate or familywise error rate—in our framework, the probability of rejecting any model would otherwise grow as the number of stimuli is increased. However, MCC in general serves to increase type-II errors (i.e., the probability of failing to reject a model that does not describe the observed responses). In the absence of a technique to directly control type-II errors (since in general the alternative hypothesis is not known), it is therefore important to ensure that type-II errors are within reasonable bounds (e.g., β < 20%; Cohen, 1992).

Within our framework, several tests are required before final classification of a cell under the model-based analysis framework. Firstly, the cell must be reliable, responsive and selective over both sets of stimuli. This ensures that extremely variable responses, which would lead to any arbitrary model passing the K–S test, will not be incorrectly classified. In addition to this criterion, when more than one model passes the K–S test, the cell is then subject to a Bayesian model comparison using likelihood ratios (see Materials and Methods). Cells with highly variable responses, or responses that do not strongly suggest one model over another, are therefore left unclassified. For the data presented here, the variability criterion ensures that only few cells are left unclassified because their responses are ambiguous under the pattern and component models (Figure 5F, “indistinguishable” bar).

We estimated the type-II error rate introduced by the K–S/MCC component of our framework, under the data presented in this work. The null hypothesis (H0) under the K–S test is that the distribution of model predicted single-trial responses is the same as the observed experimental distribution. We defined our alternative hypothesis (H1) for a given K–S rejected cell to be the model-predicted single-trial distributions, formed from the observed experimental responses. We then estimated the type-II error rate for our dataset, by performing a Monte Carlo resampling of experimental responses and model predictions under the assumption that both are normally distributed. We found that the K–S/MCC component of our framework results in a type-II error rate (β) of approximately 7% for the component model, and approximately 1% for the pattern model; both well within reasonable bounds.

If required, the statistical tests and MCC method (Kolmogorov–Smirnov and Holm–Bonferroni, in this paper) can be replaced with versions with generally higher statistical power. For example, the two-sample Anderson–Darling or Cramér–von Mises tests can have higher power than Kolmogorov–Smirnov (Razali and Wah, 2011). Similarly, Benjamini–Hochberg multiple comparisons correction might provide a better balance between control of type-I and type-II errors, as the number of stimuli increase (Verhoeven et al., 2005). Increasing the α value chosen for the MCC method will also increase statistical power, and can be used as a method to balance type-I and type-II error rates.

### Component and pattern specific responses in primary visual cortex

Neuronal responses were classified as *component*- or *pattern-classified*, first by predicting responses to plaid stimuli from the neurons' responses to individual grating components. Extending previous methods to predict neuronal responses to visual patterns with a Bayesian analysis framework allowed us to incorporate neuronal response variability to grating and plaid stimulation. By applying this model-based analysis framework we explained more than 30% of the responses of all stimulus-selective neurons during visual pattern stimulation. In a second step, we further classified the remaining neurons into *component*- and *pattern-dominant* according to their dominating response to grating or plaid stimulation. With these two steps we explained the responses of more than 70% of the total population of stimulus-selective neurons.

In addition to the 31.5% of *component-classified* cells, we found that 3% of the selective neurons in V1 were classified as pattern cells. Pattern cells have been reported to be nonexistent in the V1 of other species, such as cats (Gizzi et al., 1990), macaque monkeys (Movshon et al., 1983) and barn owls (Baron et al., 2007). Similar to our data in mouse, a range from *component*- to *pattern-selective* responses has also been described in marmoset monkey V1 (Tinsley et al., 2003). Beside these 3% of *pattern-classified* cells, we found 8% of cells responding strongly to plaid patterns but responding poorly to grating stimuli. This feature makes them impossible to classify using traditional measures based on the grating response. However, these cells were highly selective over the set of plaid stimuli; in fact, they were just as selective as *pattern-classified* cells (Figure 4C). Cells responding strongly to plaid stimuli but only weakly to grating stimuli were previously reported in area MT of New World monkeys (Solomon et al., 2011). Solomon et al. argued that these are pattern cells that receive only weak excitatory drive from stimuli having a narrow Fourier representation, such as grating stimuli (Solomon et al., 2011).

In primates, pattern integration appears to be performed at higher levels of the visual hierarchy, such as area MT (Movshon et al., 1983; Rodman and Albright, 1989; Stoner and Albright, 1992). Consistent with this tendency, a publication during review observed rare pattern responses in mouse V1 in cells with bidirectional tuning, and found more prevalent pattern responses in higher visual areas (Juavinett and Callaway, 2015). However, it is not known if there is a specific area for pattern integration in the mouse analogous to primate MT. In line with our finding of pattern integration, we have previously reported speed-tuned neurons in mouse V1 (Roth et al., 2012) and highly specific responses to artificial and natural visual scenes (Kampa et al., 2011).

The higher fraction of pattern selective cells we observed in mouse V1 might also be explained by our recording technique, two-photon calcium imaging, which provides a less biased sample of neuronal response types compared with previous studies that exclusively used electrophysiological techniques.

Interestingly, some degree of pattern integration in V1 has been reported in awake primates (Guo et al., 2004) where 9% of selective neurons were identified as classical pattern cells. It is therefore possible that the elevated percentage of pattern cells we observed could be related to differential level of anesthesia or different type of anesthetics used, or due to a species-specific reaction to anesthesia. It would be of interest to test if this proportion of pattern cells will be higher if tested in V1 in awake mice.

### Complex responses within the unclassified population

A final 26% of cells remained in our analyzed population of responsive and selective neurons, that could not be classified as either component or pattern cells (Figure 5). The lack of definitive classification of these neurons was not due to poorly tuned or weak responses—these neurons responded robustly and selectively to both gratings and plaid stimuli (Figures 1D, 2A, 5). Responses in this population were just as reliable as classified neurons (Figure 5C) and just as tuned as the pattern selective population (Figure 5D).

In previous studies, unclassified neurons were explained simply by their broad tuning (Movshon et al., 1983; Gizzi et al., 1990). However, since the majority of our unclassified population were well-tuned and reliable, the degree of stimulus tuning does not explain the presence of these neurons (Figure 5). Our stimulus protocol limited the ability to determine neuronal direction selectivity, however our analysis paradigm would not fail to classify pattern and component cells solely based on this property. It has also been shown that the degree of pattern selectivity of a neuron cannot be predicted by directional selectivity (Guo et al., 2004; Baron et al., 2007; but see Tinsley et al., 2003).

A prominent feature of responses in this population was the poor relationship between grating and plaid responses. Non-pattern, non-component neurons presented a wide range of complex responses that were not predictable by small numbers of rules operating on a single-neuron level (Figure 5B). Non-component, non-pattern neurons could therefore represent a population of cells that encode more complex visual scenes (Kampa et al., 2011).

Since existing feedforward models for visual integration suggest that two neurons with similar responses to a particular stimulus class will also have similar responses to more complex stimuli, responses within this population were not consistent with feedforward convergence of component-selective inputs.

In mice, a population of direction-selective retinal ganglion cells project to the dLGN, and some of these signals are relayed to V1 (Barlow et al., 1964; Cruz-Martín et al., 2014) suggesting the possibility that pattern cells could be formed by integrating these inputs. However, cells that integrate inputs from direction-selective RGCs would be responsive and selective to grating components, and exhibit either component-cell responses (if they suffer from the aperture problem, and respond to individual drifting grating components of a plaid); and perhaps also respond to the optimally drifting plaid, if they are able to respond to the drifting nodes formed between overlapping gratings in the plaid stimuli. However, a missing mechanism would be required to explain the facilitating pattern-selective and pattern-dominant responses exhibited by many neurons.

Consequently, our results suggest that cellular responses to complex visual patterns are dominated by network interactions, rather than feedforward influences. It is possible that feedback projections from higher visual areas contribute to the complex selectivity we observed, but considering the dominance of local inputs in terms of proportion of synaptic input, it is probable that local recurrent interactions strongly shape complex plaid selectivity (Muir and Kampa, 2012).

### Various degrees of pattern motion integration occur locally in V1

Several lines of evidence indicate that diverse degrees of pattern motion integration occur locally in V1 and are mediated by the specific micro-circuitry within this area.

Firstly, a broad range of pattern integration is visible in mouse V1. We described cells that encode individual components (*component-selective* cells) as well as neurons that responded predominantly to visual patterns (*pattern-selective* cells). But in addition to these classes, we also observed a proportion of responsive and reliable unclassified cells that could stand as intermediates in the process of plaid integration.

Simple integration of tuned responses can explain some of this variation (Figure 6). Component cell responses are intuitively built by neurons that have sharp tuning to a single orientation (Figure 6B). The response of such a component neuron to drifting gratings follows the input orientation tuning, and the neuron responds to drifting plaids that contain the appropriate grating component. A possible intermediate is shown in Figure 6C, that responds to two orientations of grating stimuli. This class of responses could be produced by neurons that integrate two narrow-bandwidth input components tuned to two different directions.

Secondly, integration of the orthogonal components of visual stimuli is reflected in the broader tuning of pattern selective cells compared with neurons that respond to the component gratings alone (Figure 4B). This can be explained by the integration of broadly tuned inputs (Figure 6D). Broad tuning of input components, combined with integration within the network, produces a broadly tuned response to drifting gratings with a peak at the preferred orientation that matches the preferred drift direction of a plaid stimulus.

Finally, pattern integration has been shown to be supralinear in primates (Rust et al., 2006). Consistent with this observation, we find *pattern-classified* cells to respond with significantly more facilitation to drifting plaids compared with the *component-classified* cells (Figure 4A). In addition, we observed a population of *pattern-dominant* cells that were both strongly facilitating (Figure 4A) and strongly selective over plaid stimuli (Figure 4C). Due to the dynamics and response saturation of calcium indicators, we cannot determine whether responses are supralinear in our experimental data. However, indicator saturation will in general reduce the effect of supralinear responses, leading to a reduction in the apparent amplitude of the strongest responses. As measured by our modulation index (MI), response saturation will tend to decrease its absolute magnitude, pushing MI toward zero. Therefore, the facilitation we observed in response to plaid stimuli would, if anything, be stronger if the firing rates of the neurons were measured directly.

The presence of significant facilitation might be explained by amplification due to highly recurrent excitatory connectivity within the sub-networks in the superficial layers of cortex (Douglas et al., 1989; Binzegger et al., 2004; Muir and Kampa, 2012) and also by dendritic mechanisms in cortical neurons leading to supralinear integration of synaptic inputs (Williams and Stuart, 2002; Gulledge et al., 2005). Response amplification has recently been shown to be an important feature of the cortical response in mouse V1 (Lien and Scanziani, 2013; Li et al., 2013a,b). Here we propose that the structure of local cortical connections might tune the selectivity of that amplification.

### Pattern motion integration by local microcircuits

The integration of visual patterns observed in mouse V1 reveals a footprint of local sensory integration by specific sub-network connectivity. The integration of different component responses into a pattern selective output has been demonstrated in converging feedforward models for primate visual cortex (Rust et al., 2006). In rodents, it has been shown that the local organization into sub-networks is tuned for integration of different sensory features (Kampa et al., 2006) (Figure 6A). Neurons composed into an inter-connected sub-network in layer 2/3 receive common input from layer 4 (Yoshimura et al., 2005) and encode collective stimulus properties such as the orientation of drifting gratings (Ko et al., 2011; Cossell et al., 2015). Multiple stimulus components could then be integrated by subsequent sub-networks to provide highly-specific responses to complex combinations of visual stimuli (Kampa et al., 2006; Muir and Kampa, 2012). This proposal is illustrated in Figure 6A. Under this scheme, two recurrent sub-networks, in layer 2/3 of V1, would receive feedforward weak orientation-tuned inputs, which are amplified through recurrent excitatory connections within each sub-network (orange and green neurons). A third excitatory sub-network shares some connections with the orange and green sub-networks, and so integrates both these component inputs with similar recurrent amplification (joint orange and green neurons). A similar connectivity scheme has been found to exist across layers in rodent neocortex (Kampa et al., 2006).

We have previously shown that neurons in mouse V1 are highly tuned to natural and artificial visual scenes (Kampa et al., 2011). While most neurons responded specifically to a particular visual scene, a minority of the neurons also encoded information about multiple scenes. Together, these findings suggest that individual components of a visual scene are encoded by specific sub-networks. Pattern integration occurs in overlapping sub-networks that receive recurrent inputs comprising several individual components (Figure 6A). Integration based on sub-network connectivity could support a wide range of response types, ranging from component to pattern cells as we show here for mouse V1. However, it is likely that this mechanism is not restricted to visual cortex since similar connectivity schemes have been found across cortical layers and in several cortical areas (Song et al., 2005; Yoshimura et al., 2005; Kampa et al., 2006; Otsuka and Kawaguchi, 2008, 2011; Brown and Hestrin, 2009; Anderson et al., 2010; Perin et al., 2011). Sub-network connectivity could therefore be a general mechanism for information integration in cortex.

### Conflict of interest statement

The authors declare that the research was conducted in the absence of any commercial or financial relationships that could be construed as a potential conflict of interest.
