# Supplementary material for: Model-based analysis of pattern motion processing in mouse primary visual cortex
Source: Front Neural Circuits. 2015 Aug 5;9:38. doi: 10.3389/fncir.2015.00038 (PMC4525018; doi:10.3389/fncir.2015.00038)
Supplement: Supplementary file 1 [file Image1.PDF]

Supplementary Material

Model-based analysis of patterned motion processing in mouse primary visual cortex

Dylan R Muir[1,2†], Morgane M Roth[1,2†], Fritjof Helmchen[1], Björn M Kampa[1,3*]

[†]Equal contribution
[1]Brain Research Institute (HIFO), University of Zürich, Zürich, Switzerland
[2]Current address: Biozentrum, University of Basel, Basel, Switzerland
[3]Current address: Department of Neurophysiology, Institute of Biology 2, RWTH Aachen University, Aachen, Germany.

*** Correspondence: Dylan R Muir and Morgane M Roth, Biozentrum, University of Basel, Klingelbergstrasse 50/70, 4056 Basel, Switzerland.**
dylan.muir@unibas.ch and morgane.roth@unibas.ch

1. Supplementary Figures and Tables

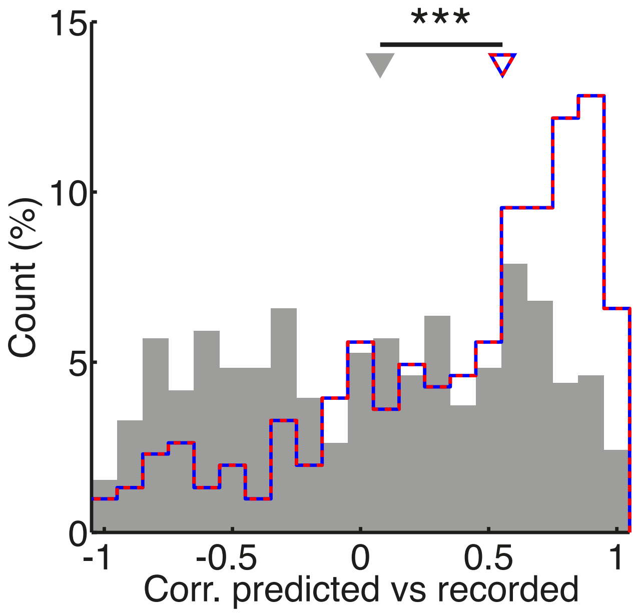

Supplementary Figure 1. Predicted responses made by our model-based analysis framework are significantly more strongly correlated with recorded responses for the set of classified neurons (dashed) than for unclassified neurons (grey) (medians 0.55 vs 0.08; $p < 0.001$, rank-sum test). For classified neurons (both *component-classified* and *pattern-classified*), the correlation was measured between the trial-averaged recorded responses and the average predicted response for the model matching the classification of that neuron. For unclassified neurons, correlations were measured between the recorded responses and both component and pattern models, then pooled.
